# Strategies to strengthen a climate-resilient health system: a scoping review

**DOI:** 10.1186/s12992-023-00965-2

**Published:** 2023-08-28

**Authors:** Ali Mohammad Mosadeghrad, Parvaneh Isfahani, Leila Eslambolchi, Maryam Zahmatkesh, Mahnaz Afshari

**Affiliations:** 1 Professor of Health policy and management, Health Economics and Management Department, Tehran University of Medical Sciences, Tehran, Islamic Republic of Iran; 2 School of Public Health, Zabol University of Medical Sciences, Zabol, Iran; 3 PhD in Health management, Health Economics and Management Department, Tehran University of Medical Sciences, Tehran, Islamic Republic of Iran; 4 School of Business and Management, Royal Holloway University of London, Egham, England; 5 School of Nursing and Midwifery, Saveh University of Medical Sciences, Saveh, Iran

**Keywords:** Climate, Climate change, Health, Health system, Resilience

## Abstract

**Background:**

Climate change is a major global threat to human health and puts tremendous pressure on health systems. Therefore, a resilient health system is crucial to enhance, maintain, and restore the population’s health. This study aimed to identify interventions and actions to strengthen a climate-resilient health system to deal with the adverse health effects of climate change.

**Method:**

This study was a scoping review. Five databases and Google Scholar search engine were searched using relevant keywords. Initially, 4945 documents were identified, and 105 were included in the review. Content thematic analysis method was applied using MAXQDA 10 software.

**Results:**

Overall, 87 actions were identified for building a climate-resilient health system and were classified into six themes (i.e., governance and leadership; financing; health workforce; essential medical products and technologies; health information systems; and service delivery). The most commonly reported actions were formulating a national health and climate change adaptation plan, developing plans for essential services (electricity, heating, cooling, ventilation, and water supply), assessing the vulnerabilities and capacities of the health system, and enhancing surveillance systems targeting climate-sensitive diseases and their risk sources.

**Conclusions:**

A holistic and systemic approach is needed to build a climate-resilient health system owing to its complex adaptive nature. Strong governance and leadership, raising public awareness, strategic resource allocation, climate change mitigation, emergency preparedness, robust health services delivery, and supporting research, are essential to building a climate-resilient health system.

## Background

Climate, derived from the ancient Greek word κλίµα, refers to the average weather conditions of a geographical area over a relatively long period of time [1]. It is the long-term (for at least 30 years) pattern of weather in a region exhibited by temperature, atmospheric pressure, wind velocity, humidity and precipitation. Climate change is the variation in the global climate over time, which can be caused by internal processes of the earth, external forces such as changes in the intensity of sunlight, and human activities. United Nations Framework Convention on Climate Change (UNFCCC) defines climate change as “a change of climate which is attributed directly or indirectly to human activity that alters the composition of the global atmosphere and which is in addition to natural climate variability observed over comparable time periods” [2].

Climate change is one of the most important challenges of the current century. It results in global temperature increases, changes in rainfall patterns, and rises in sea levels [3]. Heatwaves, sea level rise, heavy precipitation, floods and droughts are the consequences of climate change. Average climate change has been relatively stable for thousands of years. However, since the last 50 years, it has been increasing more rapidly. The average temperature of the Earth is predicted to increase by 1.8 to 4 °C by the year 2100 [4].

Climate change has a negative effect on life’s necessities such as water, food, and health [5]. It weakens the social and environmental determinants of health, including access to clean air, sufficient food, safe drinking water, and secure shelter [6]. Climate change poses a serious threat to sustainable global development. Both the drivers and the effects of climate change have severe negative impacts on people’s health. Climate change has negative effects on the sustainable development of countries and leads to a decrease in the economic growth of countries, increasing injustice in access to public services such as education and health, and even leads to conflicts and tensions between countries. The Sustainable Development Goal (SDG) 13 is to take immediate action to deal with climate change and its effects; and the SDG 3 is to ensure a healthy life and promote well-being for all people [7]. Climate and health are closely related: action on one has consequences for the other.

Climate change affects human physical and mental health directly by changing weather patterns (heat waves, droughts, floods, storms and hurricanes) and indirectly by changing the quality of water, air and food. Heat stress causes injuries and diseases such as lethargy, diarrhea, skin sensitivity, stroke and death. Air pollution increases death due to respiratory infections, lung cancer and cardiovascular diseases. About 7 million people die every year due to outdoor and indoor air pollution caused by burning fossil fuels [6]. More than 8.5 million people in Bangladesh were affected by a significant cyclone in 2007, and more than 3,500 people died [8]. Also, climate changes lead to an increase in infectious diseases transmitted through water, food and vectors and diseases caused by air pollution. A study found that dengue infection along the Texas River in the US increased by 2.6% one week after a 1 °C rise in sea surface temperature and 19.4% about 18 weeks after a 1 °C rise in sea surface temperature [9]. Natural disasters attributed to climate change can also affect a person’s mental health in the form of trauma or shock. Loss of family members, physical injury, and property damage due to natural disasters might lead to emotional trauma, fear, anger, and shock. A study in the US reported a 15% increase in suicide among rural male farmers between 2000 and 2013 during times of severe drought [10]. The World Health Organization predicted that about 5 million additional deaths due to climate change will occur in the world between 2030 and 2050 [11]. The annual direct health damage costs will be 2 to 4 billion dollars by 2030 [12].

The health system should respond to the healthcare needs of the people when facing the effects of climate change. Nevertheless, climate change threatens the health system’s capacity to protect and promote people health. Rising sea levels, typhoons, hurricanes, and floods disrupt fragile infrastructure and transportation systems, and supply materials and food [3]. These effects put tremendous pressure on the health system. Therefore, healthcare organizations may suffer due to the effects of climate change and may not be able to provide the health services needed by the people. On the other hand, healthcare organizations, due to their 24-hour activity and high energy consumption, contribute to climate change through greenhouse gas emissions. Therefore, the health system is facing the dual challenge of dealing with the human impacts of climate change and reducing its significant contribution to the carbon footprint [13]. Therefore, the health system capacity should be strengthened to respond well to the health effects of climate change.

**Fig. 1:**
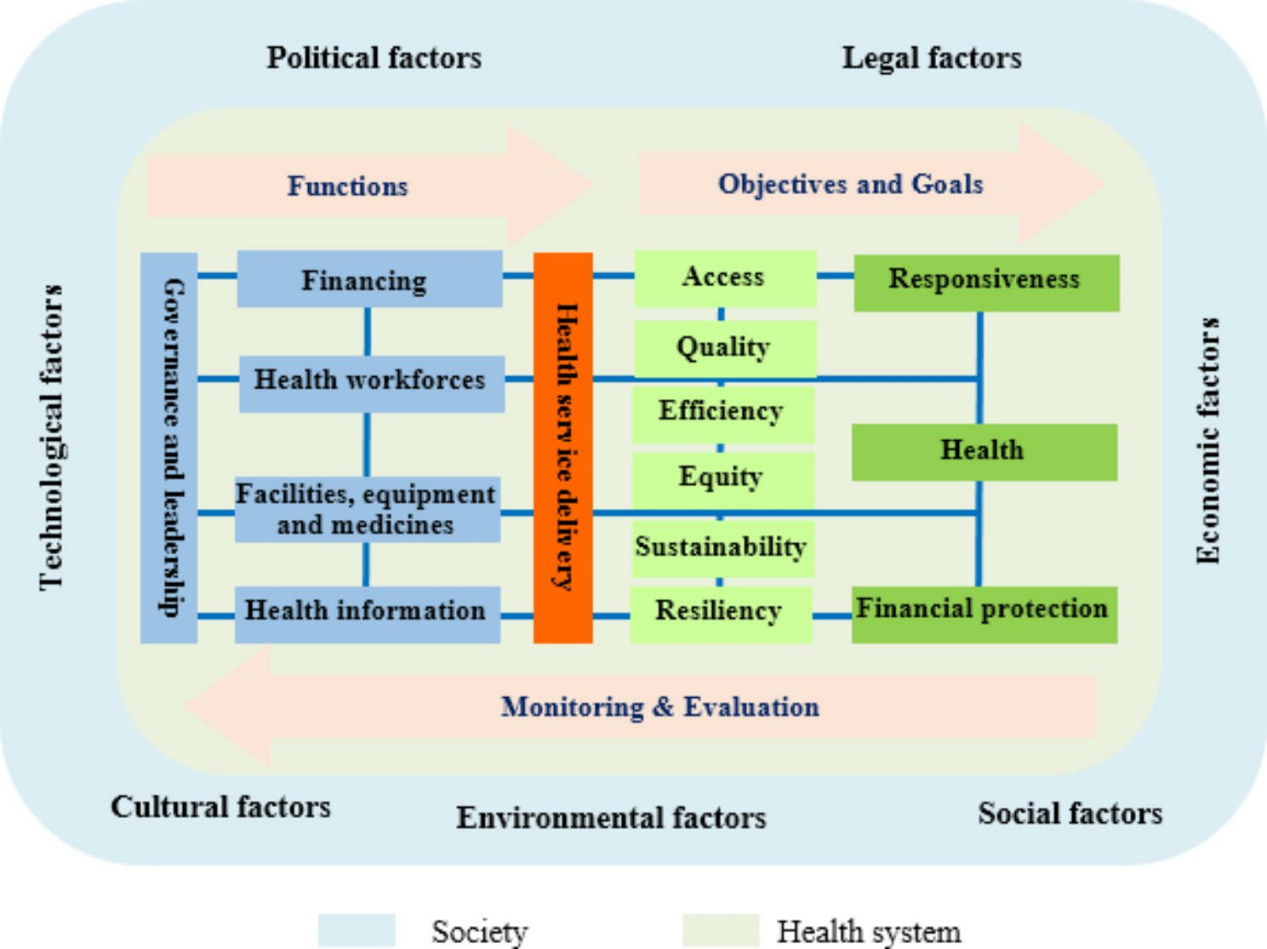
A conceptual model for health system management

The health system consists of organizations and individuals who are responsible for policy-making, financing, resource creating, and providing healthcare services to promote and maintain the health of the community, responding to people’s clinical and non-clinical expectations and protecting them from catastrophic health expenditure [14]. The World Health Organization (WHO) considers governance and leadership; financing; health workforce; medical products, vaccines, and technologies; information; and service delivery as six building blocks of the health system and necessary for achieving the goals of the health system [15]. On the other hand, political, economic, social, technological, environmental, and legal factors have a great impact on people’s health. Therefore, complex and complicated contemporary healthcare organizations [16] are surrounded by a challenging and dynamic environment [17]. Figure 1 illustrates a conceptual model of health system management. It also shows internal and external factors affecting people’s health.

Health system resilience is “the ability, capability and capacity of the health system to predict, prevent, prepare, absorb, adapt and transform when exposed to shocks and stresses and deliver routine health services continuously during the crisis management” [18]. A climate resilient health system is “one that is capable to anticipate, respond to, cope with, recover from and adapt to climate-related shocks and stress, so as to bring sustained improvements in population health, despite an unstable climate” [19]. A climate resilient health system is an opportunity for sustainable human development. It can reduce the effects of climate change on health while promoting better health. Hence, the six building blocks of the health system should be strengthened to protect and promote people health in a changing and unstable climate.

The health sector is very vulnerable to climate change. However, few policies, programs, and measures are applied, especially in developing countries, to strengthen the adaptation and resilience of the health system against climate change. Unstable and non-resilient health systems exacerbate the negative effects of climate change on people’s health. Therefore, this study aimed to identify strategies and solutions to strengthen the resilience of the health system against climate change. The findings enable health policymakers and managers to design comprehensive strategies to build climate-resilient health systems.

## Method

The scoping review method was used to conduct this research. A scoping review is secondary research to identify and analyze all the relevant literature on a topic and to map the extent, range, and nature of the literature in that specific area. The scoping review uses a systematic search method, but it does not have some of the limitations of systematic review research, such as reviewing and evaluating the quality of peer-reviewed original research articles. Therefore, scoping review is not limited to peer-reviewed literature and includes gray literature such as organizational reports, theses, abstracts of conference papers and review articles. As a result, more useful evidence is identified in a short time [20]. Arski and O’Malley’s protocol was used to conduct the scoping review, which includes six stages identifying the research question, identifying relevant studies, selecting studies to be included in the review, charting the data, collecting, summarizing, and reporting the results, and finally, consulting stakeholders [21].

The Web of Science, PubMed, Scopus, EMBASE, Scientific Information Database, and Magiran electronic databases and Google Scholar search engine were searched using the keywords climate change, climate crisis, global warming, greenhouses, health system, healthcare services, strategies, policies, guidelines, etc. and using appropriate search strategies (Table 1). The search was restricted to documents and papers written in English and Persian languages; published until June 30, 2022; and identified strategies to strengthen a climate-resilient health system.

**Table 1:** Search strategies

Databases/ search engines	Search strategy	Preliminary searches
PubMed	((“climate change“[All Fields] OR “Greenhouse Effect“[All Fields] OR “climate Change“[All Fields] OR “changing climate“[All Fields] OR “global warming“[All Fields] OR “ambient temperature“[All Fields] OR “climate crisis“[All Fields] OR “ambient temperature“[All Fields] OR (“greenhouse“[All Fields] OR “greenhouses“[All Fields]) OR (“greenhouse“[All Fields] OR “greenhouses“[All Fields])) AND “health system“[All Fields] AND (“policy“[MeSH Terms] OR “policy“[All Fields] OR “policies“[All Fields] OR “policy s“[All Fields] OR (“guideline“[Publication Type] OR “guidelines as topic“[MeSH Terms] OR “guidelines“[All Fields]) OR (“strategies“[All Fields] OR “strategies“[All Fields] OR “strategy“[All Fields] OR “strategy s“[All Fields]))) AND ((ffrft[Filter]) AND (fft[Filter]) AND (1000/1/1:2022/6/30[pdat]) AND (English[Filter] OR Persian[Filter]))	74
Web of Knowledge	((ALL=(“climate change” OR “Greenhouse Effect” OR “climate change” OR “changing climate” OR “global warming” OR “ambient temperature” OR “climate crisis” OR “ambient temperature” OR greenhouse OR greenhouse)) AND ALL=(“health system” )) AND ALL=(policies OR guidelines OR strategies) and English (Languages)	113
Scopus	ALL ( “climate Change” OR “Greenhouse Effect” OR “climate change” OR “changing climate” OR “global warming” OR “ambient temperature” OR “climate crisis” OR “ambient temperature” OR greenhouse OR greenhouse ) AND ALL ( “health system” ) AND ALL ( policies OR guidelines OR strategies ) AND ( LIMIT-TO ( PUBSTAGE, “final” ) ) AND ( LIMIT-TO ( OA, “all” ) ) AND ( LIMIT-TO ( LANGUAGE, “English” ) OR LIMIT-TO ( LANGUAGE, “Persian” ) )	3232
EMBASE	(“climate change” OR “Greenhouse Effect” OR “climate change” OR “changing climate” OR “global warming” OR “ambient temperature” OR “climate crisis” OR “ambient temperature” OR greenhouse OR greenhouse) AND “health system” AND (policies OR guidelines OR strategies)	1172
Scientific Information Database	“climate change” AND “health system” AND policies	110
Magiran	climate change AND health system AND policies	24
Google Scholar	(“climate change” OR “Greenhouse Effect” OR “climate change” OR “changing climate” OR “global warming” OR “ambient temperature” OR “climate crisis” OR “ambient temperature” OR greenhouse OR greenhouse) AND “health system” AND (policies OR guidelines OR strategies)	220 (11 pages reviewed)
**Final**		**4945**

**Fig. 2:**
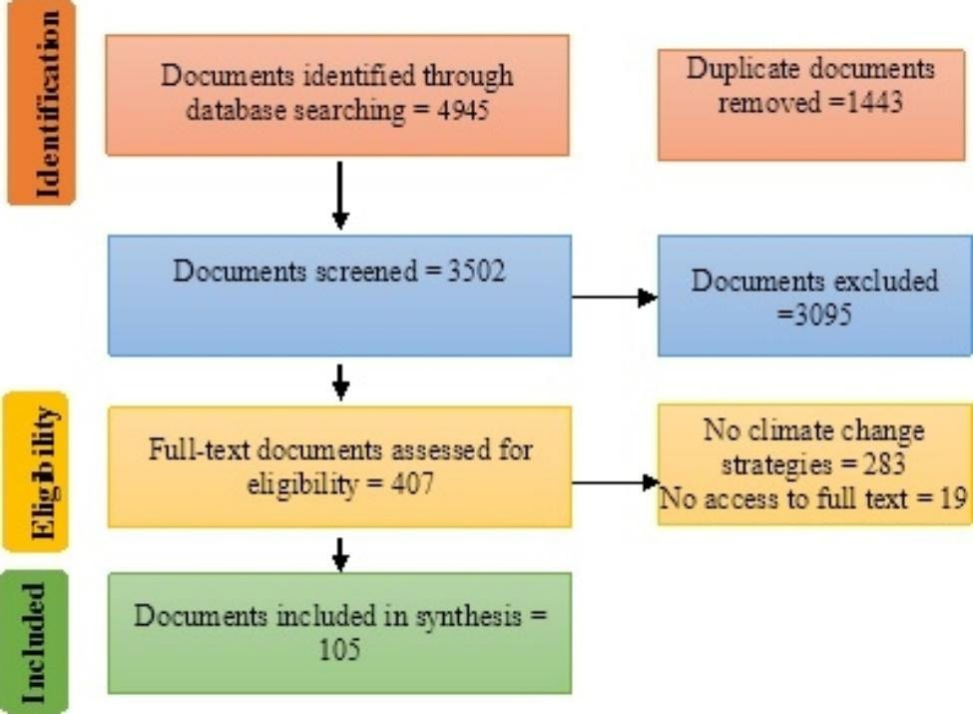
PRISMA flow diagram depicting the study selection process

The initial search resulted in 4945 documents and articles. After excluding duplicates and irrelevant articles, 3502 studies were selected for abstract examination. Then, 3095 documents were removed after reviewing their abstracts and 302 were removed after full text review. Finally, 105 articles were found eligible for inclusion in this scoping review. Figure 2 demonstrates the search process.

A data extraction form was used to collect data, which included the authors’ names, publication year, study location, journal name, study type, data collection method, and strategies to strengthen a climate-resilient health system. Braun and Clarke’s six-step thematic analysis method, including “familiarization, coding, generating themes, reviewing themes, defining themes, and preparing a report”, was used to analyze the qualitative data of this study [22]. MAXQDA software version 10 was used for data analysis. Ethical issues related to review studies were also considered.

## Results

**Fig. 3:**
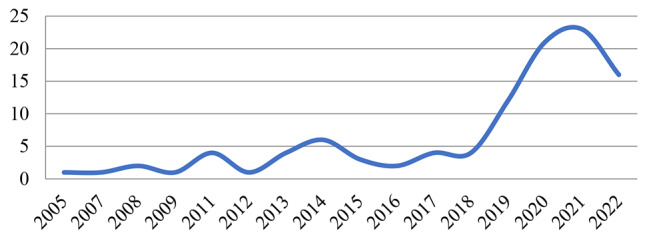
Frequency distribution of studies on policy options for strengthening a climate-resilient health system by year of publication

A total of 105 studies have investigated the strengthening of a climate-resilient health system between 2005 and the end of June 2022. Most of these studies have been conducted in 2021, 2020, and 2022 (Fig. 3).

**Fig. 4:**
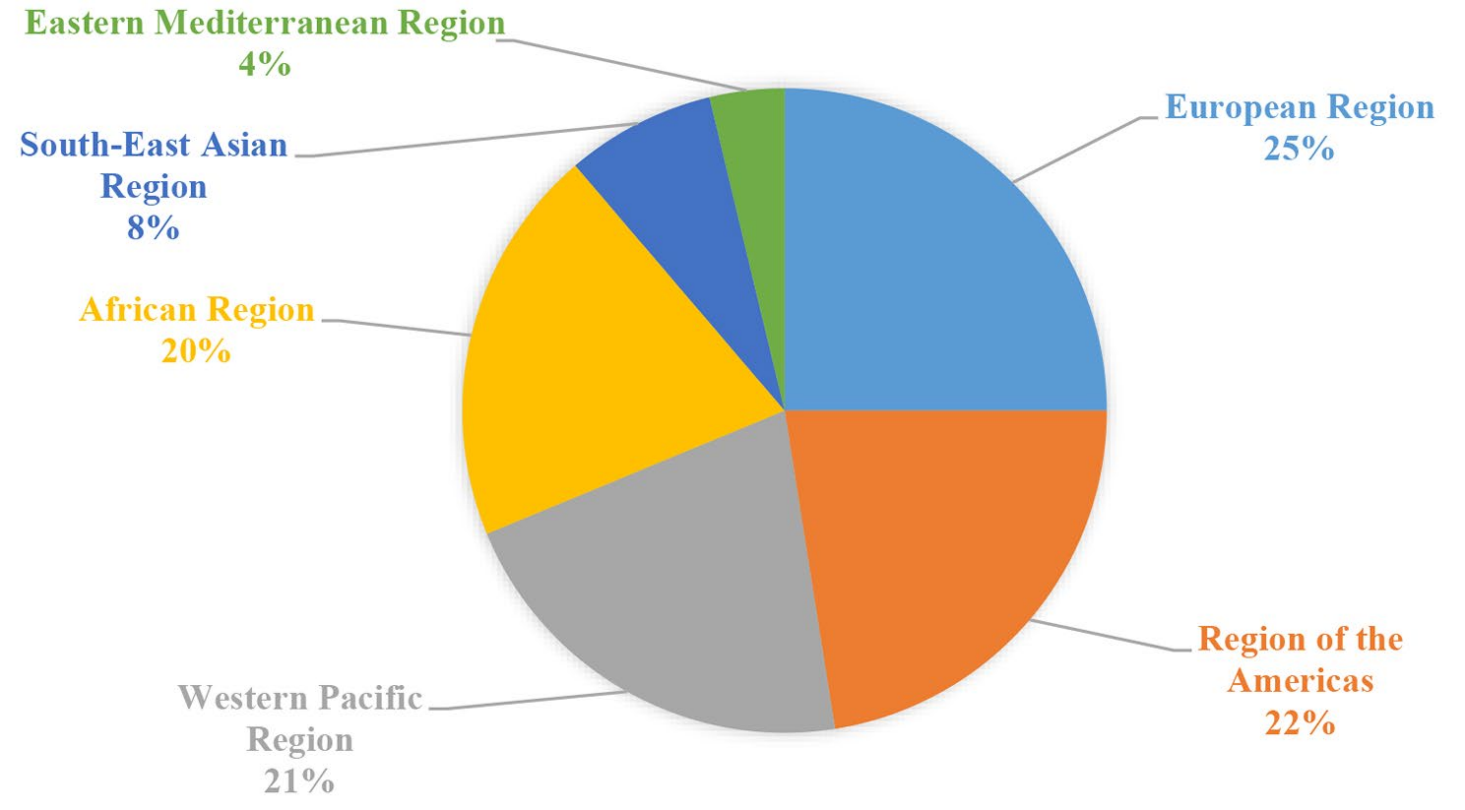
The setting of the identified papers

Studies on the strengthening of a climate-resilient health system have been performed in 6 regions of WHO (Fig. 4).

The type of reviewed documents was as follows: 38 review studies, 28 WHO reports, 22 original articles, 13 viewpoint, commentary, and editorials, 2 policy briefs or forums, 1 regional health forum report and 1 CDC report. From 22 original articles, 10 articles were done uxsing a qualitative method, 5 used quantitative study and 7 used mixed method.

Overall, 87 interventions were identified for strengthening a climate-resilient health system and were grouped into six categories, including governance and leadership; financing; health workforce; essential medical products and technologies; health information systems; and service delivery (Table 2).

**Table 2:** Strategies for strengthening a climate-resilient health system

Themes	Strategies for strengthening a climate-resilient health system (Frequency)
Governance and leadership	Developing a national health and climate change adaptation plan (10); Engaging government in health and climate change (2); Refining the regulations of the health sector to manage the health risks of climate change (2); Developing and implementing a comprehensive policy on climate change and health (1); Increasing awareness of healthcare leaders of the changing hazards (1); Designing a crisis leadership model underpinned by values and ethics (1); Using climate vulnerability indices for health planning (1); Putting pressure on governments to divest themselves of fossil fuels (1); Cooperating PHC with institutions that deal with climate change (1); Empowering key stakeholders across sectors to work at the local levels (1); Enhancing inter-sectoral and international collaboration (1); Decentralizing management through devolution of authority(1); Establishing an iterative process for managing and monitoring the health risks of climate change (1); Providing mandatory reporting on health, social, financial, and environmental performance (1); Joining an organization such as the Climate and Health Alliance (1); Designing a framework for climate change (1);
Financing	Providing adequate funding (3); Increasing research funding (2); Improving access to long-term international financing (2); Estimating the costs of action and inaction to protect health (1); Increasing funding for climate change programs in local communities (1); Increasing reliance on “cost recovery” for public sector services (1);
Health workforce	Using a sustainable and trained workforce (3); Recruiting and training of health personnel in biostatistics and epidemiology (1); Involving nursing staff in community microbiological water testing (1); Strengthening the capacity of health staff at different levels through training (1); Improving training provisions to raise awareness among professionals (1); Integrating climate change education into university training curricula (1); Engaging diverse stakeholders (1); Leading physician on climate action in everyday practice (1); Designing health educational content for residents (1); Identifying vulnerable professional groups (1); Increasing workplace awareness of infectious disease risks (1); Developing suitable protective clothing and gear (1); Spreading the knowledge and skills of health protection facing climate change (1); Ensuring access to healthcare and financial resources to support healthcare personnel working through emergencies (1);
Medical products and technologies	Planning and backup systems for essential services, i.e. electricity, air conditioner, ventilation, and water supply (9); Designing a low-carbon or net zero healthcare facility (2); Implementing infrastructure adaptations such as sustainable land use, building design, and emergency power generation (2); Allocating resources (2); Better utilization of resources (1); Improving laboratory infrastructure and testing capabilities (1); Strengthen infrastructure and equipment capacity to increase health facility resilience to natural disasters (1);
Health information systems	Forecasting climate impacts and assessing the vulnerabilities and capacities of the health system (6); Enhancing surveillance targeting climate-sensitive diseases and their risk sources (5); Research in climate change’s impact on health, and improve adaptive capacities of health services (4); Developing early warning systems on environmental risks (3); Keeping updated, centralized, easily accessible global databases on climate plans (2); Analyzing long-term multi-disciplinary climate, health, and socio-economic parameters (2); Establishing backup data systems (1); Integrating environmental, ecological, veterinary, and epidemiological data (1); Ensuring adequate data collection and data quality (1); Developing the use of proxy measures and interpolation when data may be unavailable (1); Developing spatial analysis technologies with greater integrative analysis capabilities than current GIS software (1); Increasing the ability to share data and information across jurisdictions (1); Improving the timeliness of access to laboratory testing and its results (1); Integrating health into loss and damage assessments related to climate change (1); Collating and disseminating best practices from successful countries (1); Improving communication pathways between the health sector, meteorology services, and other stakeholders (1); Capacity building for project formulation, management, and evaluation specific to climate change (1); Developing and proposing health adaptation plans (1);
Service delivery	Designing primary healthcare-based approaches to address both the immediate and long-term effects of climate change (3); Improving access to mental health services (3); Strengthening of capacity and quality for health services and facilities (2); Providing normative guidance on primary health care (2); Prioritizing vulnerable populations and geographies (2); Promoting health programs with households on efficient use of energy (2); Providing Health advisory platforms in order to improve services and education (2); Improving access to pharmaceuticals for increased health risks (2); Designing a municipal heat-wave preparedness plan (1); Accessing essential equipment, e.g., power generators and water pumps (1); Adopting energy and water efficiency programs in order to change staff behavior (1); Providing incentives to reduce energy use in buildings and transport (1); performing waste disposal under safe conditions (1); Providing information to patients about climate change and its links to human health (1); Investing in renewable energy and energy-efficient technologies (1); Minimizing unnecessary care and limiting consumables (1); Developing toolkits to reduce environmental harms from operating-room practices (1); Engaging in active relocation of vulnerable health facilities (1); Delivering an “essential services package” to the whole population (1); Strengthening chronic disease self-management programs (1); Monitoring clients in the community (1); Changing diet, e.g., eating less meat and eating from food gardens (1); Revising existing health plans to include a robust situation analysis of the climate landscape (1); Increasing resources for health emergency risk management (1); Enhancing health system capacity for rapid disease-specific emergency response (1); Updating and improving emergency risk communication strategies (1);

The most frequent interventions for strengthening a climate-resilient health system in the literature include: developing a national health and climate change adaptation plan, developing contingency plans, and backup systems for essential services, assessing the vulnerabilities, needs, and capacities of stakeholders, enhancing surveillance targeting climate-sensitive diseases and their risk sources and Research into climate change’s impact on health, and improve adaptive capacities of health services (Fig. 5).

**Fig. 5:**
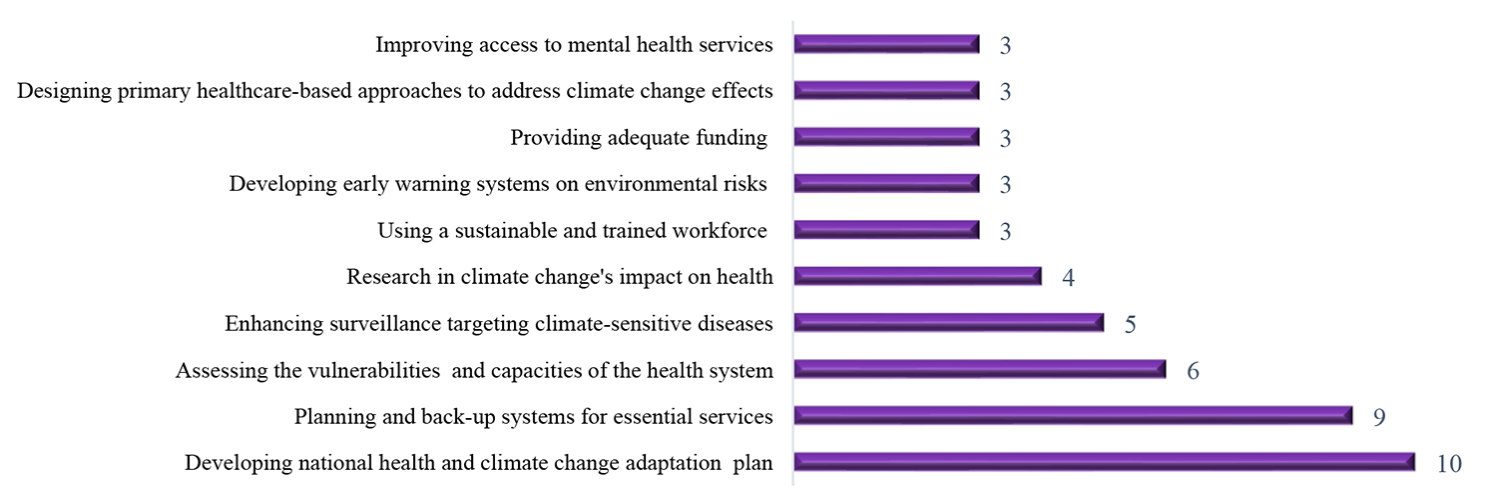
The most commonly reported interventions for strengthening a climate-resilient health system

**Fig. 6:**
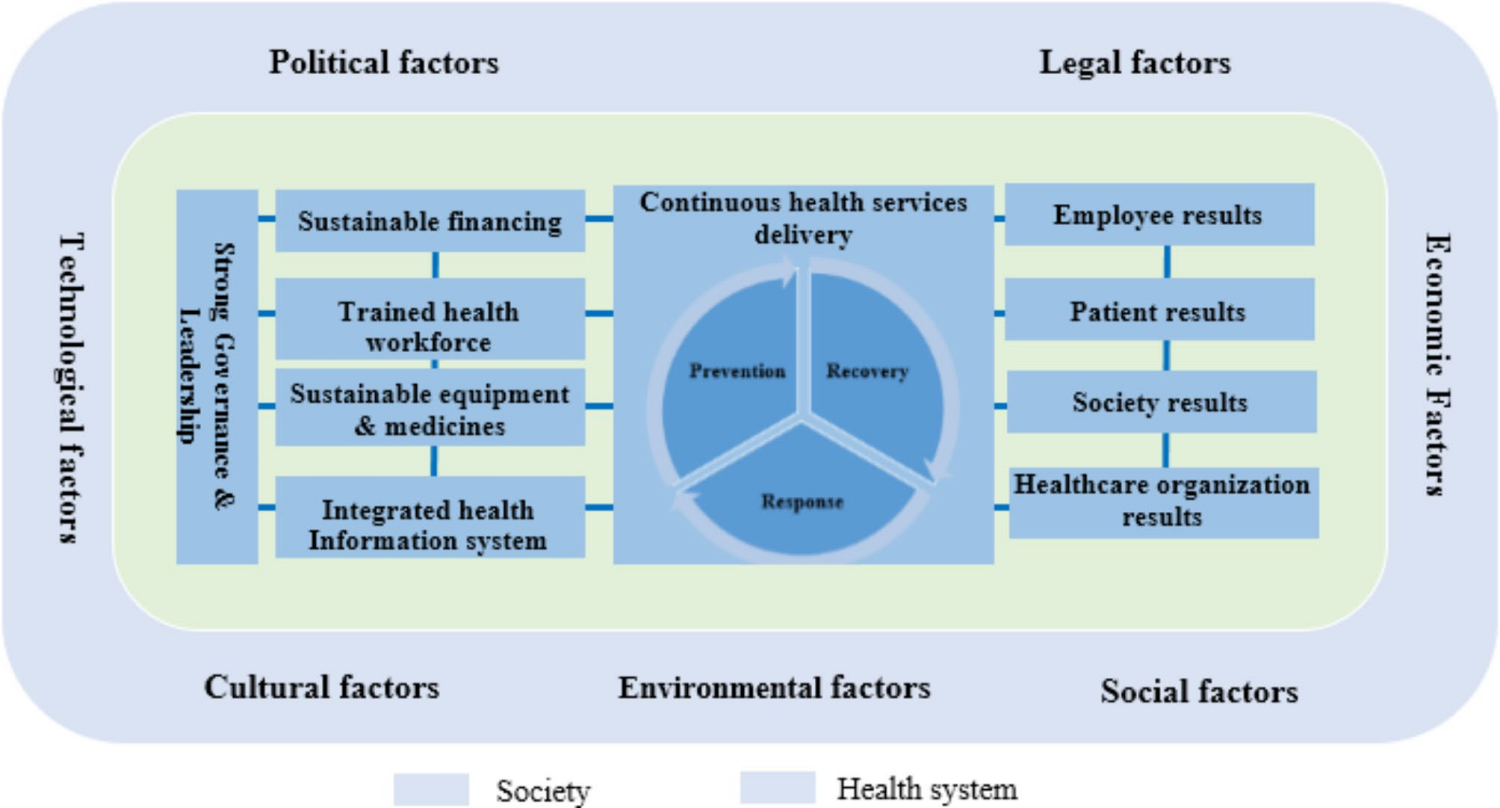
Conceptual model of a resilient health system to climate change

The six building blocks of the health system, including governance and leadership; financing; health workforce; facilities, equipment and supplies; health information system; and health service delivery processes, should be strengthened to reduce the negative effects of climate change on health care organizations, health workers, patients and the society. Furthermore, the external environment surrounding the health system, including political, economic, social, technological, environmental and legal factors, affects the performance of the health system in dealing with the negative effects of climate change, and should be considered in climate change and health programs. Figure 6 shows how a climate-resilient health system can reduce the effects of climate change on health while promoting better health for population and protect healthcare organizations and employees.

## Discussion

The main aim of this study was to identify strategies to strengthen a climate-resilient health system using scoping review method. Overall, 87 interventions (actions) were identified for building a climate-resilient health system and were grouped into six building blocks of the health system (i.e., governance, financing, workforce, equipment and medicine, information systems and health services delivery processes). The six building blocks of the health system should be strengthened against the effects of climate change.

Good governance, evidence-based policy making, strategic planning and collaboration with various stakeholders, especially with organizations affecting people’s health such as water and sewage, nutrition, energy and urban planning, are necessary to create a climate change resilient health system [13]. Governance and leadership interventions are essential to strategically manage the climate-related shocks to health systems and develop strategic health policies. Health should be considered as a priority in the national climate change policy and strategy. Therefore, many countries have developed national health and climate change policies and national health adaptation plans to address current and projected health risks. Inter-sectoral collaboration at a national and local levels should also be strengthened. However, plans’ implementations and multi-sectorial collaborations have been reported as challenging [23, 24]. Applying a “crisis leadership model” for building a climate-resilient health system was also proposed. This model focuses on planning and communication, adapting to change and updated risk assessment methods, engaging local communities and integrated early warning systems, developing a response culture, and reducing system-related greenhouse gas emissions [25].

Climate change should be considered in health policies and programs. Furthermore, political leadership is essential to address health risks of climate change in other sectors’ programs. Therefore, there is a need for greater collaboration between the health sector and other health-determining sectors, such as agriculture and urban planning, to address the root causes of climate change and its impact on health. Engaging diverse stakeholders is also a way to expand institutional capacities to provide better health services [26]. Therefore, the health sector is responsible for managing environmental determinants of health by applying “health in all policies” (HiAPs) and a multi-sectoral collaborative approach. Policy makers should consider public health in any decision they make across any policy domain. WHO designed the “One Health” initiative, which results in sustainable solutions by addressing the root causes of current and emerging problems. This integrated approach focuses on inter-sector collaboration, linking human, animal, and environmental health, and is relevant for nutrition, pollution management, the control of zoonoses, etc. As a result, it is expected to better manage global health threats [27]. Collaboration between various departments such as environmental health, disaster management, vector control, health information systems, policy making and financing are essential inside and outside the health system.

Establishing a climate change and health department at ministry of health to plan and manage climate resilient programs and develop inter-sectoral collaboration, formulate and implement the national health and climate change strategy and plan, and develop agreements between the Ministry of Health and other main stakeholders are essential to strengthen the governance and leadership of the health system.

Adequate resources and funding are required to make health systems climate resilient. The negative effects of climate change on people’s health lead to an increase in health costs. In addition, strengthening the adaptability and resilience of the health system requires a sustainable budget. Annual climate change adaptation costs in developing countries are about USD 70 billion and are projected to reach USD 140–300 billion in 2030 [28]. Therefore, financial resources should be provided to strengthen health sector resilience and support climate change and health adaptation plans. New collaborative and inter-sectoral models should be used to finance the health system. For instance, The Government of Bangladesh established the climate change trust fund in 2010 to support climate-resilient health projects [8].

Health workforce play a key role in the resilience of the health system and providing quality and effective health services. Formal education, training and mentoring opportunities on climate change health should be available to health workers to identify, prevent and manage health risks. Health workers should receive the necessary training on the effects of climate change on people’s health and the health system so that they can provide the health services when acute climate conditions occur. In addition, their capacity should be developed and improved to cope with the health threats of climate change.

It is also necessary to include climate change and health in medical education at the undergraduate, graduate, and continuing medical education levels”. Philipsborn and colleagues proposed a framework of climate change and health education content for residents to increase their professional capacity. This framework included climate change’s effects on health, its threats to healthcare delivery, and necessary adaptations in clinical practice [29]. The capacity to communicate with health workers during emergencies should be also strengthened.

Institutional capacities of healthcare organizations should be enhanced to respond effectively to climate risks. Sufficient facilities, equipment and medicines must be available to ensure the provision of health services during acute climatic conditions. Healthcare infrastructure and technologies should be climate resilient and sustainable. These can be achieved by relocating vulnerable health facilities [30], or by making infrastructure adaptations. The adaptations include sustainable land use, green and low-carbon building design, safe and sustainable purchases of materials and products, using efficient and low-carbon technologies to provide care, such as telemedicine, using renewable energies, and emergency power generation [31]. The use of medical technologies and equipment with less environmental footprint climate resiliency of the healthcare facilities.

Infrastructures and water, electricity and gas supplies of healthcare organizations may be interrupted due to extreme weather events such as floods or droughts. Therefore, the infrastructure of healthcare organizations must be built in such a way that they can withstand extreme weather events.

Health information systems play a vital role in collecting, analysing and sharing data to support decision-making by managers when facing the impacts of climate change. The health information system should be strengthened in such a way that the necessary information about the vulnerability of the health system to climate risks, the capacity of the health system to respond, and the extent of its adaptability and resilience to the effects of climate change are available to managers and health workers for evidence-based decision making [13].

Vulnerability and adaptation assessments provide information on the nature and scale of potential health risks attributed to climate change and identify both vulnerable populations and health system weaknesses [19]. Accordingly, managers can use appropriate adaptation and resiliency interventions to prevent or reduce the severity of future health impacts posed by climate change. For example, the health system vulnerability and adaptation was assessed in Vietnam from 2013 to 2017 and “the level of exposure to climate change”, “health sensitivity,” and “adaptive capacity” were reported high, high, and very low, respectively [32]. A similar assessment was carried out in Dominica and revealed numerous health consequences of climate change for the population, including infectious, foodborne, and waterborne diseases [30]. These assessments generate evidence on the capacity and climate-resilience of health system, projected changes in weather, and the population vulnerability; and inform policymakers to act urgently to reduce climate change vulnerabilities.

Climate change increases vector-borne diseases. Therefore, surveillance systems for infectious diseases should be established and developed. In addition, climate-informed early warning systems for vector-borne diseases should be developed and strengthened to minimize health effects posed by climate change. Integrated risk monitoring and early warning aim to monitor and identify changing climate and anticipate environmental risks and emergencies such as extreme temperatures or precipitation. It uses real-time information to notify the public and health experts quickly and help them be well prepared and respond effectively to prevent adverse health effects [19]. As a result, avoidable illness, injury and death can be prevented.

Different climate change exposures and related health problems occur simultaneously. Thus, several indicators affecting people’s health should be considered in early warning systems. In this regard, Linares and colleagues (2020) proposed an integrated early warning system for health in the context of climate change that includes four phases of plan activation, assessment and evaluation of health effects, actions to minimize the impact on health, and monitoring and evaluation of the plan [33].

Applied research should be conducted and their findings should be translated to practice through evidence-based decision-making. Health and climate research increases knowledge about climate conditions and their related health risks, as well as the level of global and local preparedness. It also tests new tools, technologies, and strategies and informs policymakers to develop effective policies and plans. Research should focus on the risks of climate change on health and vulnerable groups, climate-sensitivity of diseases, the design and evaluation of effective adaptation and protective measures, including public and environmental health ones, and identifying best practices to improve health care and emergency services in times of crises [32, 34].

Climate change causes different health problems, such as extreme heat stress, water and food borne diseases, allergies, mental and cardiopulmonary diseases, etc. The health sector should be aware of these effects and plan and implement climate-informed programs [17]. For example, providing health services, including health education and promotion, as well as prevention and control of common diseases and injuries, as well as locally endemic diseases, were considered in Bangladesh [35]. Maintaining access to essential primary care services like immunization and maternal health, and providing surgical, obstetric, and anesthesia services in hospitals in times of disaster are vital to promote health [36]. People expect to receive quality and effective health services from the health system [37]. Therefore, working processes in health care facilities should be strengthened so that they can provide quality health services during climate change-related disasters and extreme events.

Climate change triggers many outbreaks and emergencies. Thus, emergency preparedness and prompt crisis responses, including proactive risk management, proper risk communication, and community empowerment are required [38]. Different strategies should be applied to enhance and update emergency risk communication continuously. Community must be prepared for the risks of climate change. A prepared, active and well-organized community reduces the negative effects of emergencies and thus, reduces morbidity and mortality. Health professionals, especially primary health care workers, should educate community about the health impacts of climate change and the mitigation strategies [39]. Vulnerable groups such as the disabled and dependent people need more attention when extreme weather conditions such as heat waves or cold spells occur. They need to be contacted by health professionals and relocated to safe places [3].

Strategies and interventions should be implemented to build climate resilient and environmentally sustainable health care facilities. As a result, they can protect and improve people’s health in an unstable and changing climate and optimize the use of resources and minimize the release of pollutants and waste in the environment. However, policy makers and health managers should know that the implementation of these interventions may be associated with challenges due to structural, process and contextual limitations. High political will, good governance, coherent strategic plans, strong leadership, encouragement of internal and external collaboration and mobilization of resources are necessary.

## Conclusion

Considering the growing global trend of climate change and its adverse health effects, strengthening the health system is crucial. Due to the complex and complicated nature of health systems, a holistic approach is needed to build a climate-resilient one. Applying coherent strategies with a focus on the six building blocks of the health system will result in better health outcomes and save many lives at local and global levels. Strong governance, and leadership, raising public awareness, strategic resource allocation, climate change mitigation, emergency preparedness, robust health services delivery, and supporting research, are essential to building a climate-resilient health system. Furthermore, multi-sectoral cooperation should be enhanced. Integrating health into all policies and fostering interdepartmental collaboration with the environmental, economic, financial, energy, and education sectors is also essential.

## Data Availability

Not applicable.

## List of abbreviations

WHO: World Health Organization
UN: United Nation

## References

[1] American Meteorological Society. Glossary of Meteorology. Available at https://glossary.ametsoc.org/wiki/Climate [Access date: 07/08/2023].

[2] United Nations Framework Convention on Climate Change. Climate change. Available at https://unfccc.int/resource/ccsites/zimbab/conven/text/art01.htm [Access date: 07/08/2023].

[3] Prats EV. WHO guidance for climate-resilient and environmentally sustainable health care facilities. World Health Organization; 2020. 10.3390/ijerph17238849 PMC7731282 33260752

[4] Hansen J, Sato M, Ruedy R, Lo K, Lea DW, Medina-Elizade M. Global temperature change. Proceedings of the National Academy of Sciences. 2006;103(39):14288-93. 10.1073/pnas.0606291103 PMC1576294 17001018

[5] Costello A, Abbas M, Allen A, Ball S, Bell S, Bellamy R, Friel S, Groce N, Johnson A, Kett M, Lee M (2009). Managing the health effects of climate change. The Lancet.

[6] World Health Organization. COP24 special report: health and climate change. World Health Organization; 2018.

[7] Osborn D, Cutter A, Ullah F (2015). Universal sustainable development goals. Underst Transformational Chall Developed Ctries.

[8] Rahaman MA, Rahman MM, Rahman SH. Pathways of climate-resilient health systems in Bangladesh. Confronting Climate Change in Bangladesh: Policy Strategies for Adaptation and Resilience. 2019:119 – 43.

[9] Raffa RB, Eltoukhy NS, Raffa KF (2012). Implications of climate change (global warming) for the healthcare system. J Clin Pharm Ther.

[10] Berman JD, Ebisu K, Peng RD, Dominici F, Bell ML (2017). Drought and the risk of hospital admissions and mortality in older adults in western USA from 2000 to 2013: a retrospective study. Lancet Planet Health.

[11] World Health Organization. Quantitative risk assessment of the effects of climate change on selected causes of death, 2030s and 2050s. World Health Organization. ; 2014. Available at https://www.who.int/publications/i/item/9789241507691 [Access date 07/08/2023].

[12] World Health Organization. Climate change and health: Climate change - the biggest health threat facing humanity. https://www.who.int/news-room/fact-sheets/detail/climate-change-and-health. 2021, [Access date: 07/08/2023].

[13] Mosadeghrad AM, Shahsavani A (2023). Strengthening the resilience of Iran’s health system against climate change. Payesh.

[14] Mosadeghrad AM (2022). A practical model for health policy making and analysis. Payesh.

[15] World Health Organization (2007). Everybody’s business–strengthening Health Systems to improve Health Outcomes: WHO’s Framework for Action.

[16] Ghiasipour M, Mosadeghrad AM, Arab M, Jaafaripooyan E (2017). Leadership challenges in health care organizations: the case of iranian hospitals. Med J Islamic Repub Iran.

[17] Esfahani P, Mosadeghrad AM, Akbarisari A (2018). The success of strategic planning in health care organizations of Iran. Int J Health Care Qual Assur.

[18] Ezzati F, Mosadeghrad AM, Jaafaripooyan E (2023). Resiliency of the iranian healthcare facilities against the Covid-19 pandemic: challenges and solutions. BMC Health Serv Res.

[19] World Health Organization. Operational framework for building climate resilient health systems. World Health Organization; 2015.

[20] Neyazi N, Mosadeghrad AM, Afshari M, Isfahani P, Safi N (2023). Strategies to tackle non-communicable diseases in Afghanistan: a scoping review. Front Public Health.

[21] Arksey H, O’Malley L (2005). Scoping studies: towards a methodological framework. Int J Soc Res Methodol.

[22] Braun V, Clarke V (2006). Using thematic analysis in psychology. Qualitative Res Psychol.

[23] WHO. Health and climate change: country profile 2022: Czechia. World Health Organization; 2022.

[24] WHO. Health and climate change: country profile 2022: Bulgaria. World Health Organization; 2022.

[25] Tochkin J, Richmond J, Hertelendy A. Healthcare system leadership and climate change: five lessons for improving health systems resiliency. BMJ Lead. 2022. 10.1136/leader-2021-000583 37013877

[26] Sen B, Dhimal M, Latheef AT, Ghosh U. Climate change: health effects and response in South Asia. BMJ. 2017;359. 10.1136/bmj.j5117 29146578

[27] Ganesh C, Schmeltz M, Smith J (2020). Introduction climate change and the legal, ethical, and Health issues facing Healthcare and Public Health Systems. J Law Med Ethics.

[28] Neufeldt H, Christiansen L, Dale TW (2020). Adaptation gap report 2020.

[29] Philipsborn RP, Sheffield P, White A, Osta A, Anderson MS, Bernstein A (2021). Climate change and the practice of medicine: essentials for resident education. Acad Med.

[30] Schnitter R, Verret M, Berry P, Chung Tiam Fook T, Hales S, Lal A, Edwards S (2019). An assessment of climate change and health vulnerability and adaptation in Dominica. Int J Environ Res Public Health.

[31] Aslan M, Yıldız A (2019). How blameless are hospitals in climate change? An example of a province in Turkey. Environ Socio-economic Stud.

[32] Tuyet Hanh TT, Huong LT, Huong NT, Linh TN, Quyen NH, Nhung NT, Ebi K, Cuong ND, Van Nhu H, Kien TM, Hales S. Vietnam climate change and health vulnerability and adaptation assessment, 2018. Environmental Health Insights. 2020;14:1178630220924658. 10.1177/1178630220924658 PMC7309337 32612364

[33] Linares C, Martinez G, Kendrovski V, Diaz J (2020). A new integrative perspective on early warning systems for health in the context of climate change. Environ Res.

[34] Anderko L, Chalupka S, Du M, Hauptman M (2020). Climate changes reproductive and children’s health: a review of risks, exposures, and impacts. Pediatr Res.

[35] Rahman A (2008). Climate change and its impact on health in Bangladesh. In Health Forum.

[36] Roa L, Velin L, Tudravu J, McClain CD, Bernstein A, Meara JG (2020). Climate change: challenges and opportunities to scale up surgical, obstetric, and anaesthesia care globally. Lancet Planet Health.

[37] Mosadeghrad AM, Ferlie E (2016). Total quality management in healthcare. Management innovations for healthcare organizations: adopt, abandon or adapt.

[38] Xie E, Howard C, Buchman S, Miller FA (2021). Acting on climate change for a healthier future: critical role for primary care in Canada. Can Fam Physician.

[39] Green MS, Pri-Or NG, Capeluto G, Epstein Y, Paz S (2013). Climate change and health in Israel: adaptation policies for extreme weather events. Isr J Health Policy Res.

